# Laugh syncope as a rare sub-type of the situational syncopes: a case report

**DOI:** 10.1186/1752-1947-2-197

**Published:** 2008-06-07

**Authors:** Katsufumi Nishida, Sean K Hirota, Jinichi Tokeshi

**Affiliations:** 1 Department of Medicine, John A Burns School of Medicine, University of Hawaii, Honolulu, HI, USA; 2 John A Burns School of Medicine, University of Hawaii, Honolulu, HI, USA

## Abstract

**Introduction:**

Laughter is a good medicine; it enhances cardiovascular health and the immune system. What happens, however, if a person laughs too much or the laughter becomes out of control? Laughter-induced syncope is rare and likely goes unrecognized by many health care providers. It is thought to be another form of Valsalva-induced syncope.

**Case presentation:**

We report the case of a 56-year-old, moderately obese (body mass index of 35) man with a past medical history of sleep apnea, hypertension and hyperlipidemia who suffered from syncope secondary to intense laughter. The patient also had a history of syncope in the distant past when he collapsed on the floor for several seconds. Treadmill stress testing after the incident revealed no arrhythmia or ischemic disease, although he complained of dizziness after the test and a sudden drop in blood pressure was noted.

**Conclusion:**

Laughter-induced or gelastic syncope is extremely rare. It is thought to be a sub-type of the situational syncopes.

## Introduction

Syncope is a transient loss of consciousness and postural tone secondary to inadequate cerebral perfusion that spontaneously resolves without medical intervention. It is a relatively common clinical problem accounting for 1% to 1.5% of emergency department visits and around 6% of hospital admissions annually [1]. However, syncope remains a diagnostic challenge for clinicians, as the differential diagnosis is extensive (Table 1). Among the various classifications, neurally mediated, cardiac and unexplained etiologies appear to be the most common diagnoses. In a prospective study of 341 patients presenting with syncope, a cardiac cause of syncope was established in 23% of the patients, a neurally mediated cause in 58% and the cause of syncope remained unexplained in 18% [2].

**Table 1:** Classification of syncope

*Cardiac*
Aortic stenosis, hypertrophic cardiomyopathy, pulmonary embolism, aortic dissection, myocardial infarction, left atrial myxoma, cardiac tamponade, atrioventricular block, sick sinus syndrome, tachyarrhythmia, bradyarrhythmia
*Neurally mediated (reflex mechanisms)*
Vasovagal, situational (micturition, laughter, tussive, defecation, postprandial, sneeze, swallow), orthostatic syncope, carotid sinus syncope
*Neurologic*
Transient ischemic attack, subclavian steal syndrome, Takayasu disease, seizure
*Metabolic*
Hypoxia, hypoglycemia, hyperventilation
*Psychiatric*
Panic disorder, conversion reaction, hysteria
*Drug-induced*
Vasodilators (nitrates, calcium channel blockers, angiotensin-converting enzyme inhibitors), phenothiazines, antidepressants (tricyclic agents, monoamine oxidase inhibitors), central nervous system depressants (barbiturates), drugs associated with torsades de pointes (quinidine, procainamide, disopyramide, amiodarone, sotalol, flecainide), diuretics, digitalis, insulin, marijuana, alcohol, cocaine
*Unknown origin*

Laughter-induced or gelastic (derived from the Greek word for laughter, 'gelos') syncope is extremely rare. It is a sub-type of the situational syncopes hypothesized to be the result of a neurally mediated reflex triggered by increased intrathoracic pressure. Intense laughter causes repetitive forced expirations in a staccato pattern with a Valsalva-type effect. The associated increase in intrathoracic pressure reduces venous return resulting in decreased cardiac output and a transient reduction in cerebral perfusion. It has also been proposed that strenuous laughter might produce isometric muscle contraction resulting in acute vascular dilatation, thereby exacerbating the reduction in venous return [3].

Normally the body is able to compensate for these changes through cerebral vascular autoregulation and autonomic reflexes. In one of the most well-known reflex arcs, reduced cardiac output leads to decreased stimulation of carotid sinus and aortic arch baroreceptors, as well as mechanoreceptors in the left ventricle wall [4]. The resulting increase in sympathetic tone maintains blood pressure for adequate cerebral perfusion. However, in neurally mediated syncopes, there is acute and inappropriate hypotension and bradycardia exacerbating the reduction in cerebral perfusion, resulting in a transient loss of consciousness. It is hypothesized that increased ventricular contraction in response to reduced venous return stimulates the left ventricle mechanoreceptors to a degree that is able to override the baroreceptor reflex and cause an inappropriate increase in parasympathetic tone [4]. Aside from laughter-induced syncope, this mechanism is also thought to account for syncope secondary to coughing, sneezing and other Valsalva-related activities.

To the best of the authors' knowledge, only five cases of laugh syncope among adults have been reported in the literature [3,5-8] (Table 2). We present the case of a 56-year-old man who suffered from syncope secondary to intense laughter.

**Table 2:** Reported cases of laugh syncope among adults

Reference	Age (years)	Sex	Predisposing factor	Recurrence
[3]	62	Male	Brachiocephalic stenosis	Yes (prior to stent placement)
[5]	32	Male	None	No
[6]	60	Male	Hypovolemia	No
[7]	63	Male	None	Yes
[8]	55	Male	None	No

## Case presentation

A 56-year-old, moderately obese (body mass index of 35) man with a past medical history of sleep apnea, hypertension and hyperlipidemia presented to his primary care physician for routine health maintenance. He mentioned that he had recently been very busy with overtime work, which had left him exhausted. He informed the physician of an incident that occurred one evening as he entertained his colleagues in a fine restaurant. While waiting for the meals to be served, a guest had told a very amusing joke and the patient began to laugh heartily, "Ha, ha, ha, ha... " in decrescendo until he was out of breath. To everyone's surprise, he then fell forward resting his head on the table and remained unresponsive for a few seconds before regaining consciousness. Prior to losing consciousness, he described feeling short of breath and noted that his surroundings were becoming dark. No seizure-like activity or incontinence was witnessed. After the episode he denied nausea, diaphoresis or otherwise feeling sick and proceeded to eat when his entrée was served. The remainder of the evening was without incident.

He had a history of syncope in the distant past in which he had collapsed on the floor for several seconds following hours of overtime work. Treadmill stress testing after the incident revealed no arrhythmia or ischemic disease, although he complained of dizziness after the test and a sudden drop in blood pressure was noted. At the time, he had been in poor physical condition with a sedentary lifestyle. He was encouraged to exercise and remained free of symptoms until this episode.

## Discussion and conclusion

Laugh syncope was diagnosed in the patient based on his characteristic presentation. Situational syncopes such as laugh syncope are usually diagnosed using history from the patient [9]. In the other known cases of laugh syncope reported in the literature, more thorough and extensive diagnostic evaluations were performed [3,5-8]. However, all cases had a common characteristic history of transient loss of consciousness following intense laughter, which potentially could have obviated the need for an expensive diagnostic evaluation. The burden of syncope evaluation on the health care budget is significant. It has been estimated that hospital admission of patients presenting with syncope for inpatient evaluation costs the health care system more than US$2.4 billion dollars per year in the USA [10].

To date, numerous causes of syncope have been recognized (Table 1). This presents a diagnostic challenge to clinicians. In the initial evaluation of syncope patients, the diagnostic rate is estimated to be only 20% to 50% [1]. Even after extensive diagnostic work-up, no cause can be identified in 15% to 30% of patients [1]. There is no diagnostic 'gold standard' for syncope. A careful history, physical examination and electrocardiogram (ECG) are usually the most efficacious means used to establish a diagnosis or determine the need for further diagnostic testing [11]. Unlike history and physical examination, ECG actually has a low diagnostic yield [1]. It should be included in the initial syncope evaluation, however, because it is noninvasive, relatively inexpensive and can detect potentially life-threatening conditions. Extensive laboratory and imaging studies rarely provide useful diagnostic information unless specifically indicated. Unfortunately, the evaluation of patients presenting with syncope is frequently unable to reveal a clear etiology and many cases remain unexplained.

With syncope patients, emergency physicians are often confronted with the difficult decision of whether the patient should be admitted for inpatient evaluation and management. Syncope patients are frequently admitted following an initial non-diagnostic evaluation because of concerns of underlying life-threatening conditions (for example, dysrhythmias, pulmonary embolism or acute coronary syndrome) or belief that inpatient evaluation will reveal the cause [12]. So the question of who should be hospitalized for syncope remains. As noted previously, the diagnostic yield of extensive work-up is relatively poor. The focus in syncope evaluation, therefore, has shifted from attempting to make a specific diagnosis to risk stratification. Through careful risk stratification, it is hoped that health care resources will be more efficiently allocated to those patients most at risk of serious outcome and, hence, more likely to benefit from inpatient care. In a recent update of their 2001 clinical policy on syncope, the American College of Emergency Physicians emphasized the use of history, physical examination and standard 12-lead ECG to risk-stratify patients and admit those with risk factors for adverse outcome such as heart failure, coronary artery disease, structural heart disease, older age, concurrent comorbidities, hematocrit less than 30% (if obtained) or abnormal ECG [1].

Several other studies have also developed clinical decision rules to aid in the risk stratification of syncope patients. The San Francisco Syncope Rule (SFSR) has been shown to be sensitive in identifying patients at risk of serious outcome within 7 days of initial emergency department presentation based on the following predictors: abnormal ECG, shortness of breath, systolic blood pressure under 90 mm Hg, hematocrit less than 30% and congestive heart failure by history or examination [13]. However, another external validation cohort found that the SFSR had a lower sensitivity and specificity than reported previously suggesting that the rule may require further validation before it can be applied safely in clinical practice [14].

The prognosis for patients presenting with syncope varies according to the underlying etiology. One population-based study found that cardiac and neurologic syncope were associated with an increased risk of death from any cause and an increased risk of cardiovascular events and stroke, respectively [15]. By comparison, patients with vasovagal, orthostatic, medication-induced and situational syncope had no increase in the risk of death from any cause compared with patients without syncope [15]. Although laugh syncope was not specifically addressed in this study, as a type of situational syncope, we extrapolate that its prognosis is likely benign. To the best of our knowledge, no study to date has investigated the prognosis of laugh syncope.

## Abbreviations

ECG: electrocardiogram; SFSR: San Francisco Syncope Rule.

## Competing interests

The authors declare that they have no competing interests.

## Consent

Written informed consent was obtained from the patient for publication of this case report. A copy of the written consent is available for review by the Editor-in-Chief of this journal.

## Authors' contributions

KN and SKH drafted the manuscript, JT cared for the patient, performed the investigation that led to the patient's diagnosis and assisted in the formulation of the manuscript. All authors read and approved the final manuscript.
